# Ultradian Cortisol Pulsatility Encodes a Distinct, Biologically Important Signal

**DOI:** 10.1371/journal.pone.0015766

**Published:** 2011-01-18

**Authors:** Andrew McMaster, Maryam Jangani, Paula Sommer, Namshik Han, Andy Brass, Stephen Beesley, Weiqun Lu, Andrew Berry, Andrew Loudon, Rachelle Donn, David W. Ray

**Affiliations:** 1 Endocrine Sciences Research Group, Manchester Academic Health Sciences Centre, University of Manchester, Manchester, United Kingdom; 2 The Arthritis Research UK Epidemiology Unit, Manchester Academic Health Sciences Centre, University of Manchester, Manchester, United Kingdom; 3 Faculty of Life Sciences, Manchester Academic Health Sciences Centre, University of Manchester, Manchester, United Kingdom; 4 School of Computer Science, Manchester Academic Health Sciences Centre, University of Manchester, Manchester, United Kingdom; 5 School of Biological Sciences, University of Kwa-Zulu, Durban, South Africa; Pennington Biomedical Research Center, United States of America

## Abstract

**Context:**

Cortisol is released in ultradian pulses. The biological relevance of the resulting fluctuating cortisol concentration has not been explored.

**Objective:**

Determination of the biological consequences of ultradian cortisol pulsatility.

**Design:**

A novel flow through cell culture system was developed to deliver ultradian pulsed or continuous cortisol to cells. The effects of cortisol dynamics on cell proliferation and survival, and on gene expression were determined. In addition, effects on glucocorticoid receptor (GR) expression levels and phosphorylation, as a potential mediator, were measured.

**Results:**

Pulsatile cortisol caused a significant reduction in cell survival compared to continuous exposure of the same cumulative dose, due to increased apoptosis. Comprehensive analysis of the transcriptome response by microarray identified genes with a differential response to pulsatile versus continuous glucocorticoid delivery. These were confirmed with qRT-PCR. Several transcription factor binding sites were enriched in these differentially regulated target genes, including CCAAT-displacement protein (CDP). A CDP regulated reporter gene (MMTV-luc) was, as predicted, also differentially regulated by pulsatile compared to continuous cortisol delivery. Importantly there was no effect of cortisol delivery kinetics on either GR expression, or activation (GR phosphoSer^211^).

**Conclusions:**

Cortisol oscillations exert important effects on target cell gene expression, and phenotype. This is not due to differences in cumulative cortisol exposure, or either expression, or activation of the GR. This suggests a novel means to regulate GR function.

## Introduction

Glucocorticoids undergo a circadian oscillation, with cortisol levels in humans peaking in the early morning and subsequently decreasing to low levels in the evening [1]–[5]. These diurnal fluctuations arise from signaling between the hypothalamic suprachiasmatic nucleus and the adrenal gland, and consist of both the autonomic nervous system and hormonal regulation of the HPA axis [6], [7]. Additionally, ultradian rhythms of cortisol exist in healthy human volunteers, with a pulse of production every 1–2 hours [8], [9]. These secretory episodes occur with a constant frequency, but a variable amplitude, allowing for the production of the subsequent circadian rhythm [10]–[16].

Glucocorticoids mediate their effects through the intracellular glucocorticoid receptor (GR). There are rapid effects on intracellular signaling kinases [17], and effects on target gene transcription [18]–[22]. The activated GR can either bind directly to target DNA sequences, or via a tethering mechanism to other, DNA-bound transcription factors, including NFkB, AP-1, HNF4, C/EBP and ets [18], [23], [24]. There is evidence that activated nuclear receptors, including GR, are highly dynamic within living cells, suggesting a “hit and run” mechanism for target gene regulation, coupled to a highly ordered cycle of chromatin remodelling [19], [25]–[27]. The intrinsic temporal dynamics of this system exist with constant ligand exposure, but the impact of temporal fluctuations in ligand availability, likely the situation seen in-vivo, is unknown [11], [28].

Recent studies have revealed unexpected, rapid, ligand-driven changes in GR-DNA interactions, with consequences for target gene transcription. Such changes were only seen with low-affinity, endogenous GR ligands, and not with the higher affinity synthetic ligands typically used to probe GR function in-vitro [29]. These studies suggest that the evolutionary conservation of glucocorticoid pulsatile release has consequences for the biologically accurate expression of patterns of genes [29].

Glucocorticoids are used extensively as anti-inflammatory agents but are difficult to use long-term because of serious metabolic side-effects (ie hypertension, hyperglycaemia, hyperlipidaemia, osteoporosis, gastric ulceration, glaucoma). Current oral maintenance glucocorticoid treatment, used for multiple chronic inflammatory diseases, delivers one large dose of a synthetic glucocorticoid with a prolonged biological half-life.

In this study we use flow through cell culture to deliver pulsed and continuous cortisol to HeLa cells. Gene expression profiling and bioinformatic analysis shows differential expression and indicates that the temporal kinetics of cortisol delivery significantly alters the cellular response.

## Materials and Methods

### Flow-through culture system

A flow-through cell culture system with integrated cell culture chamber was designed. The system was driven using a peristaltic pump (Watson-Marlow Bredel Pumps), maintained in a 37°C incubator. An externally controlled pinch valve allowed the flow of medium to be switched between alternative medium reservoirs, allowing the temporal delivery of compounds without perturbation to the target cells.

#### Pulse modelling

Recording cortisol levels: The flow-through cell culture system was optimised to replicate endogenous cortisol pulses. A single, complete pulse was performed using the flow-through system. 361 ng/ml (1 µM) cortisol medium was infused for 10 or 20 minutes, followed by switching to non-cortisol containing medium container for 50 mins. The flow rate was 14 ml/h. Samples were collected every 5 minutes from the effluent tube and cortisol levels were measured using a cortisol ELISA system (R&D Systems).

### Treatment schedules

Approximately 1×10^6^ HeLa cells were seeded into the flow-through culture dish and left for 24 hrs (100% confluent). Cells were washed with PBS. 5 mls of FBS-free flow-through medium with bicarbonate levels optimised for buffering with air was added and the dish sealed. Untreated control: One input reservoir contained flow-through medium (RPMI 1640+10% FBS). A continuous 14 h treatment was given using the flow-through medium alone.

### Pulsatile cortisol treatment

The two input reservoirs contained either flow-through medium or flow-through medium with 361 ng/ml (1 µM) cortisol. The cells were given pulses of the cortisol medium for 10 minutes followed by a 50 minutes washout period. This was repeated twelve times, at hourly intervals, followed by a 2 hr washout period using the flow-through medium. Infusion of cortisol containing medium for 10 minutes gave approximately 200 ng/ml cortisol peaks in the cell culture vessel.

### 100 ng/ml cortisol continuous treatment

The two input reservoirs, one containing serum free flow-through medium, and the other flow-through medium with 100 ng/ml (277 nM) cortisol. A continuous 12 hour treatment of 100 ng/ml was followed by a 2 hr washout period using the flow-through medium alone. This delivered the same total amount of cortisol to the target cells over 12 hours as the continuous pulsatile delivery system, and was therefore a matched cortisol dose control.

### 200 ng/ml cortisol continuous treatment

The two input reservoir contained either flow-through medium or flow-through medium with 200 ng/ml (554 nM) cortisol. A continuous 12 hour treatment of 200 ng/ml was followed by a 2 hr washout period using the flow-through medium alone. The 200 ng/ml continuous treatment equated to the concentration at the peak of the pulse.

Therefore, all the cells received flow-through medium, and the same rate was used for all studies.

The 200 ng/ml treatment was used for the microarray experiments only.

### Cell proliferation assay

HeLa cells were plated, and cultured as per the treatment schedules described above. After 14 hours conditioning viable cells were counted, by trypan blue exclusion, using a haemocytometer by a masked observer.

### HeLa cell apoptosis assay

Cells were seeded at 2×10^6^/ml overnight. Adherent HeLa cells were exposed to pulsatile (100 ng/ml), continuous (100 ng/ml) or control for 12 hrs, followed by a final 2 hrs cortisol-free washout period. The cells were then washed with PBS and labelled with APC conjugated-Annexin V and analysed with FACS.

### RNA extraction

Total RNA was extracted using RNeasy Mini Kit (Qiagen). RNA quality was checked using the RNA 6000 Nano Assay, and analyzed on an Agilent 2100 bioanalyser (Agilent Technologies). RNA was quantified using a Nanodrop ultra-low-volume spectrophotometer (Nanodrop Technologies). RNA samples required a 260∶280 nM ratio of >1.9 to be analysed further.

### cDNA synthesis

Approximately 100 ng total RNA was used to synthesize cDNA. Synthesis was carried out using a Two-Cycled cDNA Synthesis Kit (Affymetrix). A GeneChip Sample Clean Up Module kit (Affymetrix/QIAGEN) was used for cDNA cleanup. The final elution step resulted in approximately 12 µl cDNA. Quality of the cDNA was checked using the RNA 6000 Nano Assay and analyzed on an Agilent 2100 Bioanalyser.

### Biotinylation and fragmentation of cRNA

Biotin labeling of cRNA was carried out using Genechip IVT labeling kit (Affymetrix). 12 µl of cDNA was used and the resultant cRNA was purified using the GeneChip Sample Clean-up Module with a final elution volume of 19 µl in RNase free water. cRNA was quantified using the Nanodrop spectrophotometer. 15 µg cRNA was used for fragmentation. The reaction was carried out in 5X fragmentation buffer at 94°C for 35 min.

### Hybridization

Affymetrix HG U133A chips were hybridized with the labeled cRNA, and then scanned, as previously described [30].

### Array analysis

Details are contained in the Supplementary Materials S1.

### Transcription factor activity informatics

Modeling and visualising transcription factor (TF) networks was based on the principles of Xie et al.[31] and of Sanguinetti et al.[32]. This utilises both predicted transcription factor binding sites (TFBS), transcription factor abundance, and our microarray based gene expression data. Further description of the methods used are provided in the Supplementary Materials S1.

### Real-time qPCR

qRT PCR was carried out according to manufacturers instructions. Details are provided within the Supplementary Materials S1.

### Luciferase reporter gene assay

HeLa cells (2×10^6^) were plated and transfected with 2 µg mouse mammary tumour virus-luciferase (MMTV-Luc) plasmid and 0.1 µg Renilla luciferase plasmid using FUGENE transfection reagent (Roche) according to the manufacturer's instructions. Cells were exposed to either control (normal flow through medium, no cortisol), continuous flow with cortisol 100 ng/ml or dose-equivalent pulsatile cortisol (total amount delivered 100 ng/ml). After 12 h, a cortisol-free washout period of 2 hours was performed. Cells were washed twice in PBS, lysed, and MMTV-Luc and Renilla-Luc activity measured using the dual luciferase reporter gene assay. Relative luminescence units were calculated.

### Immunoblot analysis of GR

Cells were lysed in radioimmunoprecipitation assay buffer (RIPA) buffer (50 mM TrisCl pH 7.4, 1% NP40, 0.25% Na-deoxycholate, 150 mM NaCl, 1 mM EDTA, and Complete protease inhibitor (Roche), with phosphatase inhibitor cocktail 1 and 2 (Sigma)) and complete protease-inhibitor cocktail. Protein concentration was determined using the Bio-Rad Bradford assay reagent. Whole cell lysates (75 µg protein) were resolved by SDS-PAGE, blotted, and membranes incubated with primary antibodies (Mab GR - BD Transduction Labs, and P-GR (Ser211) – Cell Signalling Technologies). Membranes were subsequently stripped and blotted for α-tubulin to confirm equal loading of samples and transfer of protein, as described before [17], [30]. Immunoblots analysis was performed on two separate cell incubations.

### Statistical analysis

Where comparisons between two groups are made unpaired Student's t test was used, and where comparison was between more than two groups initial analysis was by ANOVA, followed by post hoc Bonferroni t test.

## Results

### Cortisol pulse modelling

**Figure 1:**
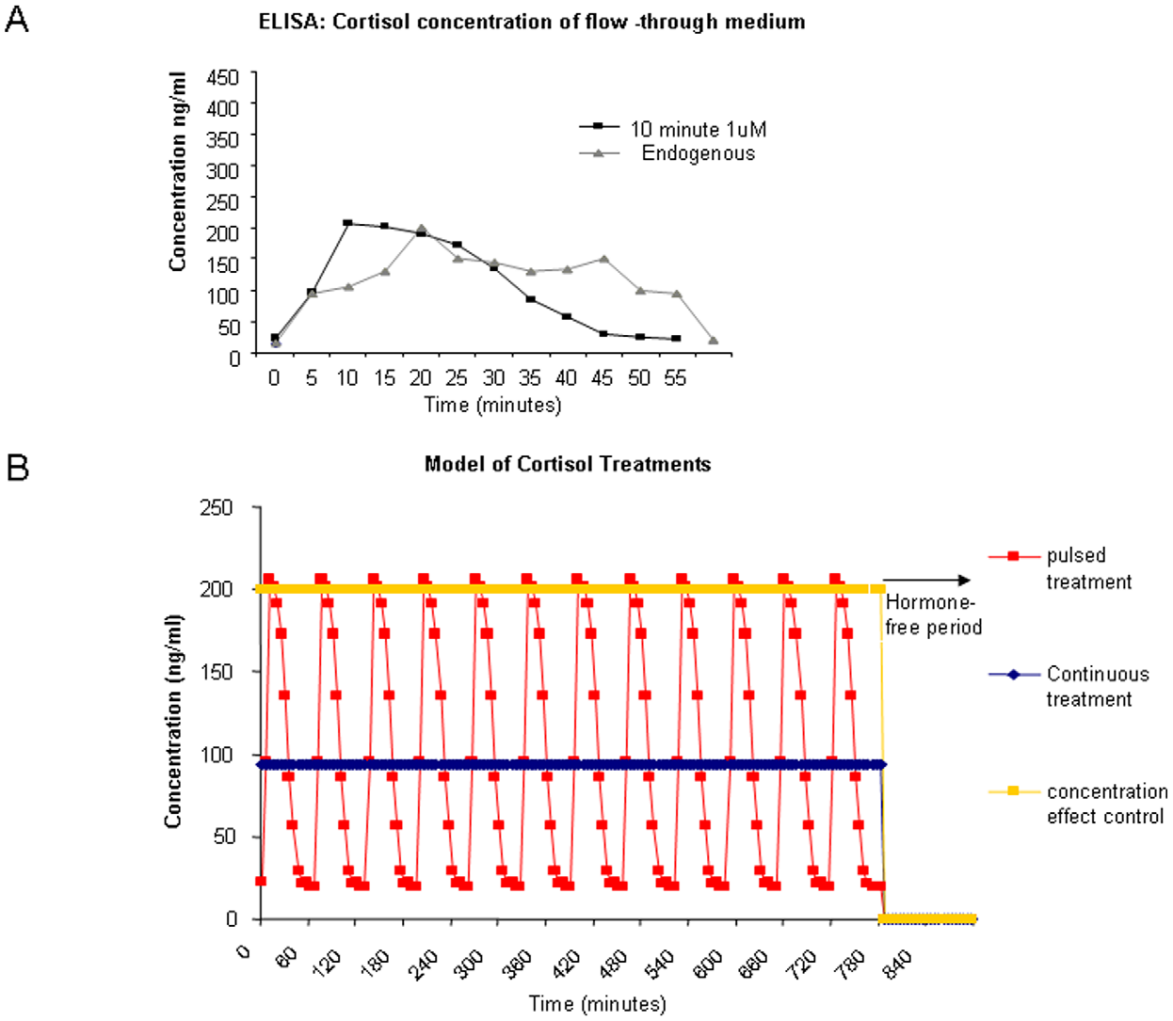
Establishment of flow through culture system to analyse pulsatile glucocorticoid action. (A) Pulse modeling using cortisol ELISA. The cell chamber was infused with cortisol containing flow-through medium for 10 minutes (Black), and then the input reservoir was switched to medium without cortisol for 50 mins. Effluent medium was collected every 5 minutes for ELISA analysis. Endogenous rat corticosterone concentrations are plotted (Grey) are re-plotted for comparison [13] (B): Simulated data for the various cortisol treatment schedules: Pulsed treatment (200 ng/ml cortisol every hour), continuous 100 ng/ml cortisol and continuous 200 ng/ml cortisol. After 12 hours, a cortisol-free washout period of 2 hours was performed. Cells were then harvested for analysis.

A cortisol ELISA was used to calibrate the flow through system to replicate endogenous cortisol pulses (Figure 1A). The culture dish was infused with the cortisol flow-through medium for 10 or 20 minutes, and then input was switched to the cortisol-free flow-through medium for 50 minutes. An endogenous rat pulse of corticosterone, as measured by the automated frequent blood sampling technique developed by Lightman [11], [14], is included on the graph as a reference. The 20 minute infusion of cortisol medium resulted in a peak cortisol concentration in excess of 350 ng/ml. However, the cortisol medium for 10 minutes, followed by a 50 minute washout period, produced a pulse that closely replicated the endogenous corticosterone rhythm seen in-vivo in frequently sampled rats.

In order to investigate the effect of temporal cortisol levels on target cell response appropriate treatment schedules were designed. The dosing schedules were; untreated control, continuous treatment, pulsatile treatment, and concentration control (Figure 1B). After each treatment schedule cell analysis was performed or RNA was extracted from the HeLa cells for microarray analysis. In all cases a 2 h washout period was used to avoid the acute impact of final cortisol concentration.

#### Cell proliferation and 3viability

**Figure 2:**
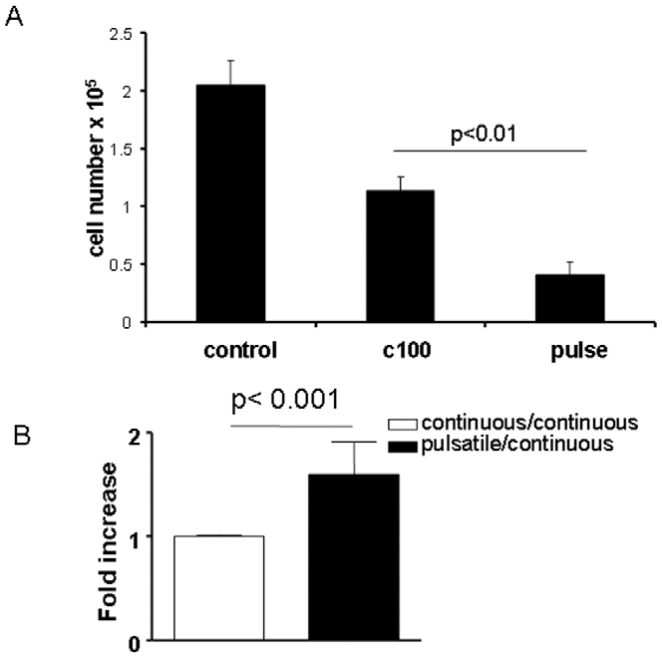
Glucocorticoid delivery kinetics affect target cell proliferation, and apoptosis. (A) Glucocorticoid regulation of cell proliferation. HeLa cells were seeded at 2×10^6^ per well and subjected to either control (normal flow through medium, no cortisol), continuous flow with cortisol 100 ng/ml, or dose-equivalent pulsatile cortisol. After 16 hours cells were counted using a haemocytometer. The experiment was performed in triplicate, bars indicate mean and SEM. Comparison was by ANOVA, and post hoc Bonferroni t test * indicates p<0.01. (B) Effects of pulsatile and continuous hydrocortisone (Hc) on induction of apoptosis in HeLa cells. Cells were seeded at 2×10^6^/ml overnight. Adherent HeLa cells were exposed to pulsatile (100 ng/ml), continuous (100 ng/ml) or control (no Hc) for 12 hrs. The cells were then washed with PBS and labelled with APC conjugated-Annexin v and analysed with FACS. Data shown are the relative fold increase in induction of apoptosis by pulsatile treatment compared to the continuous Hc. Graph is mean of n = 3 experiments. Comparison was by ANOVA followed by post hoc Bonferroni t test * indicates p<0.001.

Initial assessment of the effects of cortisol dynamics on HeLa cell proliferation and viability was carried out. Pulsatile cortisol caused a significant reduction in live cell survival compared to continuous exposure, reflecting a complex cell response to cortisol delivery kinetics (Figure 2A).

To determine the contribution of apoptosis to the variation in cell viability observed HeLa cells were exposed to the identical treatment regime as used for the cell viability assay, and then Annexin V staining determined by FACS. This confirmed that the reduction in cell viability and cell proliferation observed was due, at least in part, to increased apoptosis (Figure 2B).

#### Glucocorticoid target gene profiling

In order to further explore the effect of cortisol dynamics on target cell response we used an unbiased strategy of expression gene profiling. An overview of the analysis plan for the resulting microarray data is shown in Figure S1.

**Figure 3:**
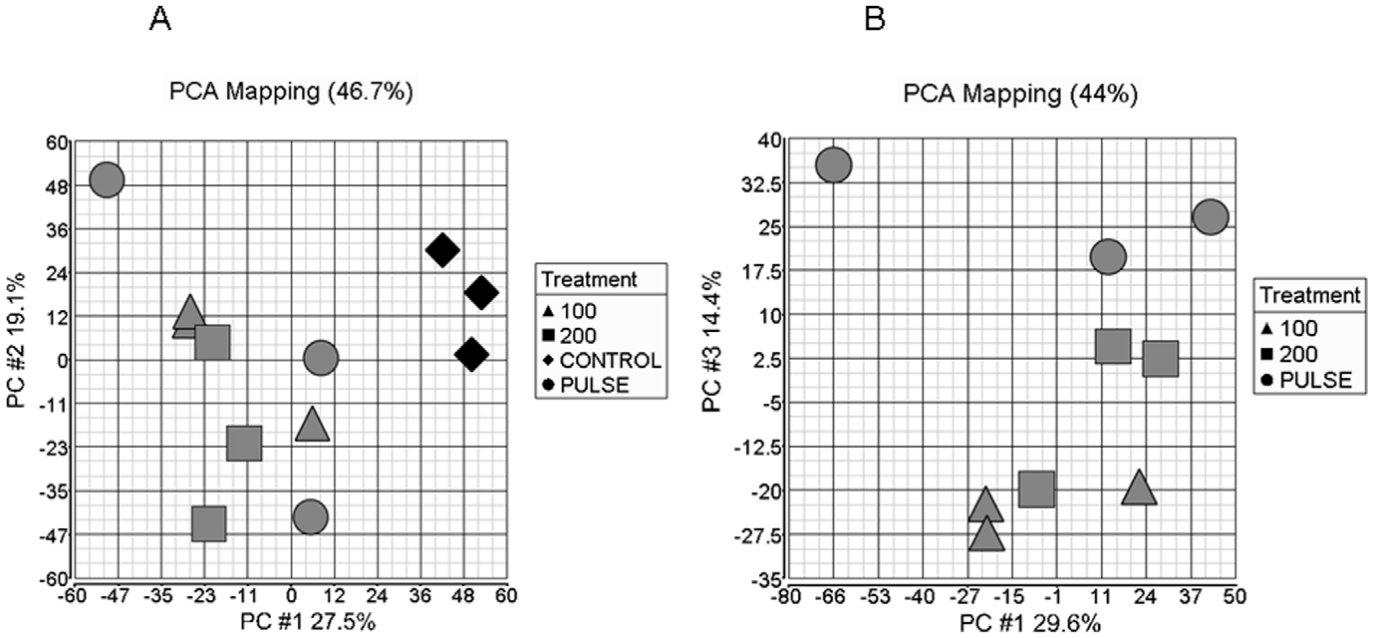
Microarray analysis of the transcriptome response to pulsatile versus continuous glucocorticoid treatment. (A) Principal components analysis (PCA) of microarray data to establish relationships between the samples using all genes in the microarray dataset. Control, pulsatile cortisol delivery (PULSE), continuous 100 ng/ml (100), and continuous 200 ng/ml (200) are indicated on the plots. There is a clear separation between all the cortisol treatments and control in the first component. (B) When cortisol treatments are considered separately pulsatile treatment segregates from the two continuous treatments in the third component.

Global gene expression changes in our dataset were explored by principal components analysis (PCA) to reveal the relationships between the different cortisol delivery regimes [33]. Plotting array samples in PCA space shows that cortisol treatment has a major effect on the gene expression profile (Figure 3A). The first principal component (x-axis; PC#1) accounts for the largest component of gene expression change in the dataset, and therefore the biggest differences between arrays. This first component showed that pulse treatment separates from control in a similar way to 100 ng/ml (C100) and 200 ng/ml (C200) treatment (Figure 3A). However, when PCA is done without the control treatment, the pulse treatment replicates separated from both continuous treatments on component 3 (Figure 3B; y axis, PC#3). Taken together these results indicated that pulse treatment was largely similar to C100 and C200 treatments, but that pulse delivery does cause weaker but identifiable gene expression differences from continuous delivery.

A detailed description of the analysis strategy for the microarray data is given in Supplementary Materials S1. We used DAVID software to group the regulated genes according to function [34]. This gene classification was done on the pool of all 469 differentially expressed genes. Significant over-representation of genes associated with cell adhesion (Benjamini corrected p-value 6.8×10^−4^) was found, as well as mitotic cell cycle (Benjamini corrected p-value <0.1); graphically presented in Fig. S2.

**Figure 4:**
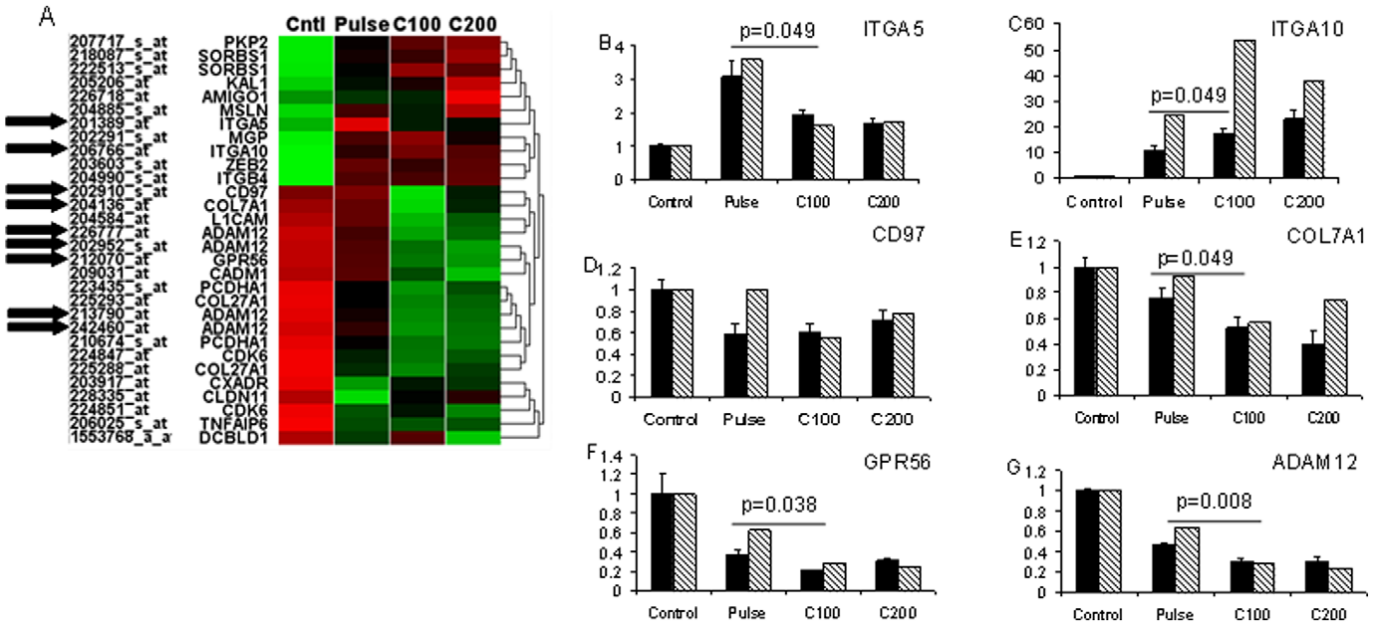
Gene ontology analysis of the transcriptome response to pulsatile versus continuous glucocorticoid treatment. (A) Cortisol regulated genes with a differential response to pulsatile versus continuous 100 ng/ml delivery were analysed by DAVID gene ontology software. Probe set, gene abbreviation, heat map of microarray expression and hierarchical clustering are shown. Red and green indicate positive and negative deviation from mean, respectively, with the intensity of colors representing the extent of deviation. Cluster transcripts levels of >5% were required to include a particular biological process or pathway. Black arrows indicate genes validated by qRT-PCR. (B–G) Quantitative RT-PCR validation of microarray data. Confirmation of the microarray results were sought using expression analysis of the indicated genes by quantitative real time PCR. All expression values were normalised to the average of β-actin and GAPDH. Data represents the mean and standard error of biological triplicate experiments. Data was analysed by unpaired Student's t test, exact P values are shown. qPCR data is indicated by solid bars while array data is indicated by hatched bars.

The cluster heat map of cell adhesion genes affected by the mode of cortisol delivery is shown in Figure 4A. Of the six genes selected, ITGA5 was upregulated more by pulsatile delivery of cortisol, compared to continuous, and COL7A1, GPR56 and ADAM12 were all significantly repressed to a greater extent by continuous cortisol compared to pulse (Figure 4B-G). The expression of ITGA10 was increased more by continuous than pulsatile cortisol, and the expression of CD97 was down-regulated, by all cortisol treatments (Figure 4 B-G). In addition to these cell adhesion genes, the expression of an additional nine genes from other functional groups was assessed by qRT-PCR (Fig. S3) to validate array results. All of these genes were shown to be regulated by glucocorticoids and for three of these (the GR, NR3C1; NR6A1 and FKBP5), there was a significantly different effect seen between C100 and pulsatile delivery.

MAOA was predicted by the microarray to be induced to a greater extent by continuous cortisol, and this was seen by qRT-PCR, though it did not reach statistical significance, and FOXO1 was induced to a similar extent by both pulsatile, and continuous cortisol in both the array, and the qRT-PCR. MAP3K7 was predicted by the microarray to be repressed to a greater extent by pulsatile cortisol, and this effect was not confirmed by qRT-PCR measurement.

#### Transcription factor analysis and validation

**Figure 5:**
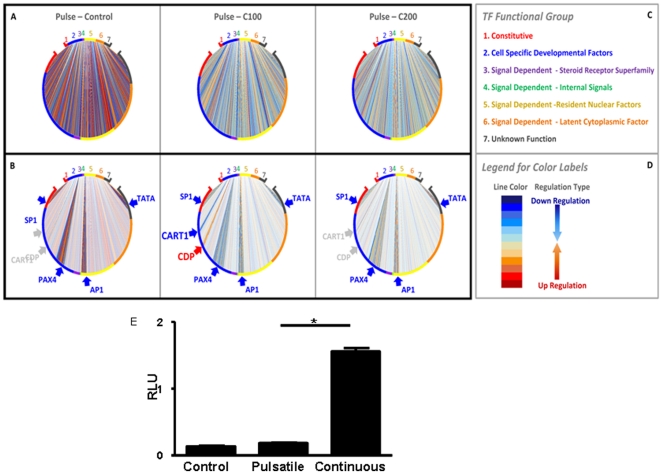
Analysis of possible transcription factor regulatory networks driven by pulsatile versus continuous glucocorticoid treatment. (A) The significantly different (SigDiff) transcription factor activities (TFAs) in the continuous cortisol treatment (100 ng/ml) (CCT) compared with the pulsatile cortisol treatment (PCT) (of matched dose) are presented in the limpet-like plots. A line presents a SigDiff TFA between the strengths of the TFA of the PCT and the CCT. If a SigDiff is greater than zero, it is displayed in bluish color and indicates that the TFA for the TF-gene pair is significantly higher in the PCT (Down-regulation in the CCT); while, if a SigDiff is less than zero, it is displayed in reddish color and indicates that the TFA for the pair is significantly higher in the CCT (Up-regulation in the CCT). (B) A break-down view for the 6 TFs showing the biggest differences between PCT and CCT (especially C100). To highlight the SigDiff TFAs for the 6 TFs, other SigDiff TFAs are dimmed out. (C) The perimeters of the plots are broken into different coloured regions corresponding to the different functional groups which are listed in the key. (D) The legend for line color. (E) Experimental validation of transcription factor analysis. 2×10^6^ HeLa cells were plated and transfected with 2 µg mouse mammary tumour virus-luciferase (MMTV-Luc) plasmid and 0.1 µg Renilla luciferase plasmid. Cells were exposed to either control (normal flow through medium, no cortisol), continuous flow with cortisol 100 ng/ml or dose-equivalent pulsatile cortisol (total amount delivered 100 ng/ml). After 12 hours, a cortisol-free washout period of 2 hours was performed. Cells were washed twice in PBS, lysed, and MMTV-Luc and Renilla_Luc activity measured using the dual luciferase reporter gene assay. Relative luminescence units were calculated. Comparison between the continuous and pulsatile exposures was by unpaired Student's t test. The graph is representative of two independent experiments, each performed in triplicate. ** indicates p<0.001.

A novel strategy for modeling and visualising transcription factor (TF) networks that utilises predicted transcription factor binding sites (TFBS), transcription factor abundance, and our microarray based gene expression data was employed (Fig. S4). This predicted differential activity of several transcription factors including SP-1, and CDP (Figure 5 A-D). CDP was of interest as a regulator of the mouse mammary tumor virus (MMTV), which is also known to be glucocorticoid responsive [35]. Importantly, pulsatile and continuous cortisol exerted quite different effects on MMTV promoter activity, as predicted (Figure 5E).

#### Immunoblot analysis of total GR and phospho-GR

**Figure 6:**
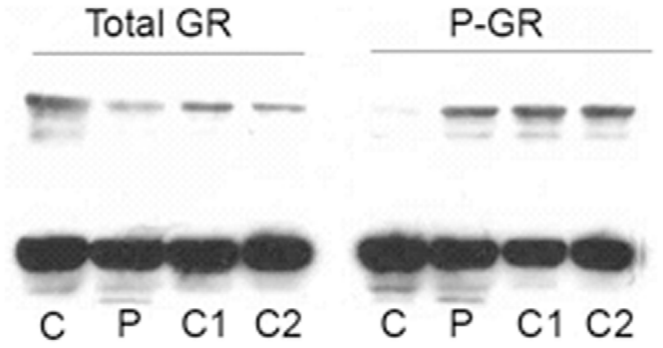
Expression of total GR and Ser 211 phosphorylated GR (P-GR) in HeLa cells after different flow-through schedules. Cells were set up and treated with control (C), pulse (P), c100 (C1), & c200 (C2) treatment schedules, as for the microarray experiments. Whole cell extracts were prepared and immunoblotting was performed using the indicated antibody.

As the principal mediator of glucocorticoid action is likely to be the GR itself, and expression of the GR gene (NR3C1) was differentially affected by delivery kinetics, the expression level of the GR protein was assessed. There was no apparent difference in the ligand dependent repression of GR expression seen under any of the experimental conditions observed (Figure 6). Moreover, the induction of phosphorylated Ser^211^GR also was similar (Figure 6).

## Discussion

The existence of pulsatile release of glucocorticoids from the adrenal glands, and the attendant pulsatility of serum levels have been known for some time [13]. However, the downstream implications of such short-term fluctuations in serum concentration for glucocorticoid action on target tissue have not been explored. Indeed, therapeutically, there has been a move towards longer acting glucocorticoids to facilitate once daily dosing as a way of improving patient compliance. In this current work, we have sought to explore the biological consequences of short-term fluctuations in glucocorticoid concentrations for the target cell response.

Detailed physiological studies have generated high resolution plots of varying glucocorticoid concentrations over time, with an estimated inter pulse period of 80–120 mins [36]. Our study design replicated pulse characteristics in-vitro. A cortisol conditioning period followed by a hormone free wash-out period was adopted This had the advantage of conditioning cells with either pulsed or continuous glucocorticoid but the hormone free wash out period avoided the potential confounding factor of the different final concentration of cortisol in the cell culture medium. Initial experiments demonstrated significant effects of the cortisol dynamics on HeLa cell proliferation and apoptosis. Principal components analysis (PCA) of the microarray data, with separation of control samples from cortisol treatments, indicated that the main patterns of gene expression responses were similar regardless of cortisol delivery regime. PCA in the absence of control samples showed separation of pulsed delivery samples from low and high dose continuous cortisol treatments. This implies that the pulse mode of cortisol delivery exerts an impact on the pattern of gene regulation in target cells.

The objective of the microarray analysis was to identify networks of genes whose expression were differentially regulated by the different modes of glucocorticoid delivery, and the analysis strategy is outline in Fig. S4. No genes passed our initial strict criteria for differential expression between the different cortisol regimes, although a large number did in the comparisons to control. Compared with the control, cortisol treatments showed differential regulation of genes involved in cell adhesion, cell cycle, and cytoskeleton. Employing less stringent criteria for differential gene expression between pulse and continuous deliveries it was found that genes involved in cytoskeleton and cell adhesion were influenced by pulse delivery. Significant differences in gene expression were also seen using qRT-PCR. It is relevant that cytoskeleton, and cell adhesion genes are known to be targets for Gc action, as previously reported [37]. The differences discovered here are predicted to alter the final impact of Gc on cell phenotype, and provide an attractive molecular mechanism to explain the altered impact of Gc on cell proliferation, and apoptosis presented in figure 2.

Transcription factors differentiating between the delivery modes of cortisol further were also identified in silico, and one, CDP, was robustly validated experimentally.

Recent work has discovered that pulsatile delivery of the rodent physiological glucocorticoid, corticosterone, results in rapid oscillations of GR on and off target genes. These cycles correlate with pulses of gene transcription, or “gene pulsing”[29]. This suggests that naturally occurring pulses of glucocorticoid delivery have an important regulatory effect on expression of the target cell genome. In these studies continuous corticosterone delivery was compared against the same concentration given in pulses, with a resulting reduction in the cumulative glucocorticoid dose. This design differs importantly from our current work, in which the conditioning effect of pulsatile, and concentration matched cortisol cumulative doses were achieved. It is striking that in the earlier work continuous corticosterone consistently had a greater effect on target gene induction, and subsequent protein accumulation compared to pulsatile delivery [29]. We now show that dose-matched pulsatile delivery of cortisol exerts a greater effect on expression of some target genes, and a lesser effect than continuous cortisol on others, reflecting perhaps differences in the GR-target DNA sequence interaction.

A major determinant of the glucocorticoid response is the expression level of the GR. We found no significant differences in total GR protein expression, or in the activation of GR, as measured by GR phosphorylation of Ser^211^, in response to pulsatile or continuous glucocorticoid exposure.

Differential effects of pulsatile delivery on target gene expression may be related to an underlying mechanism controlling GR cycling on target regulatory elements. For instance, Stavreva et al. has described fluctuations in glucocorticoid concentration to be followed by a rapid response of the activated GR cycling on and off target gene regulatory elements [29]. Furthermore, it is likely that DNA sequence differences, taken with the wider context of the regulated gene, may affect the on and off rates for GR binding, and as a consequence make some genes more critically dependent on ligand bound GR than others. Indeed, very recent work has discovered significant GR binding to the genome even in the absence of ligand binding, and changes in GR binding to the genome dependent on corticosteroid concentration, underlining the complex relationship between ligand binding and target gene recognition and regulation [38].

In summary, our data define a role for rapid fluctuations in glucocorticoid concentration on target gene regulation, and provides a possible biological impact for the physiological fluctuations in serum glucocorticoid concentrations. We describe an important frequency modulation “FM” signal encoded by pulsatile adrenal release of glucocorticoids capable of delivering an additional level of information to target cells. This has implications for therapeutic glucocorticoid drug design and also for the administration of glucocorticoid drugs.

## Supporting Information

Supplementary Materials S1 Analysis strategy, and methodology applied to gene expression analysis.
The supplementary materials detail the analytical approach, and the methods employed to profile, and validate gene expression profiles in cells subjected to either pulse, or continuous glucocorticoid.
The initial results of such analysis are presented, with additional qRT-PCR validation.
The primer sequences used for qRT-PCR are also presented.
(DOC)

Figure S1 **Gene expression array profiling: Flow chart showing data analysis strategy.**
Initial review of the data used gcRMA, then initial analysis was by Limma and q value, and principal component analysis. Two thresholds were applied for more detailed analysis, a strict, and a more lenient one. Resulting gene lists were analysed by DAVID for functional networks, and then by hierarchical clustering, and volcano plotting.
(TIF)

Figure S2 **Volcano plots showing the magnitude of change in gene expression between pulse and continuous (C100) treatments.**
Genes expressed more highly under pulse conditions are shown to the right of the “y” axis, which depicts the (-Log_10_) of the P value. Cell adhesion genes (a – in blue) and cell cycle genes (b – in blue) that are differentially expressed between the two treatments are indicated.
(TIF)

Figure S3 **Quantitative RT-PCR validation of microarray data.**
Confirmation of the microarray results were sought using expression analysis of the indicated genes by quantitative real time PCR. All expression values were normalised to the average of β-actin and GAPDH. Data represents the mean and standard error of biological triplicate experiments. Exact P values are shown, analysis by Student's t test.
(TIF)

Figure S4 **Overview of our systematic approach.**
The data analysis pipeline is composed of five parts: (1) RMA normalization of microarray data is performed, and a binary matrix containing connection topology is constructed. (2) The microarray data and connectivity data are utilized to infer TFAs and TFCs. (3) Once TFAs are estimated, the statistically SigDiffs are calculated. (4) SigDiff TFAs and TFs are analyzed and classified into structural groups and functional groups. (5) The SigDiff TFAs and TFCs are illustrated with TF-perspective views that show TFAs with associated TFCs by TF functional group and experimental condition and round limpet-like plots which show the TFA between individual TF and genes.
(TIF)
