# Supplementary material for: Ultradian Cortisol Pulsatility Encodes a Distinct, Biologically Important Signal
Source: PLoS One. 2011 Jan 18;6(1):e15766. doi: 10.1371/journal.pone.0015766 (PMC3022879; doi:10.1371/journal.pone.0015766)
Supplement: Supplementary Materials S1 — Analysis strategy, and methodology applied to gene expression analysis. The supplementary materials detail the analytical approach, and the methods employed to profile, and validate gene expression profiles in cells subjected to either pulse, or continuous glucocorticoid. The initial results of such analysis are presented, with additional qRT-PCR validation. The primer sequences used for qRT-PCR are also presented. (DOC) [file pone.0015766.s001.doc]

**Supplementary Material:**

**Material and Methods:**

**q-RT-PCR:** The quantitative real-time PCR was carried out in 96-well qPCR plates on an ABI PRISM 7300 detector (Applied Biosystems). The primers were designed using DNAMAN (Lynnon, Canada) version 4.15 software. Primers were synthesized commercially (Eurogentec, Seraing, Belgium). The optimization and validation of primers were performed using standard ABI protocols. The sequence and optimal concentrations of primers together with the size of products are shown in Table 1.

The real-time PCR was performed in a final volume of 25 µl consisting of optimal concentration (10ng) of reverse transcribed cDNA mixed with optimal concentrations of primers and qPCR Cyber green Master mix kit (Eurogentec, Belgium), using a standard amplification profile. All samples were performed at least in triplicate.

qPCR data analysis: Human ß-actin and GAPDH were used as reference genes. Relative quantitation values were expressed using the 2^–Ct^ method as fold changes in the target gene normalized to the reference gene (average of ß-actin and GAPDH) and related to the expression of the untreated controls, as previously reported (1;2).

**Microarray analysis.**

Technical quality control was performed with dChip (V2005) (www.dchip.org), using the default settings. Background correction, quantile normalization, and gene expression analysis were performed using gcRMA in Bioconductor (3;4). Principal component analysis (PCA) was performed with Partek Genomics Solution (version 6.0, Copyright 2005, Partek Inc., St. Charles, MO, USA). Differential expression analysis was performed using Limma using the functions lmFit and eBayes (4). Gene lists of differentially expressed genes were controlled for false discovery rate (fdr) errors using the method of QVALUE (5).

A gene list of differentially expressed genes (631 probe sets) was created by filtering for probe sets with a q-value less than 0.1 and fold change greater than ±1.75. Functional annotation of the genes was performed using DAVID version 2 (6). Probe sets of genes belonging to the same Gene Ontology category were hierarchically clustered (probeset means for each treatment were standardised (mean set to zero and standard deviation set to one) prior to clustering using maxdView software (available from <http://bioinf.man.ac.uk/microarray/maxd/>)). Microarray data has been submitted in a MIAME compliant standard to the Array Express database (Experiment E-MEXP-2447, <http://www.ebi.ac.uk/microarray-as/ae/>).

**Transcription factor activity informatics.**

We developed a novel strategy for modeling and visualizing transcription factor (TF) networks, which utilizes both knowledge of how TF binding site (TFBS) regulate gene transcription and sets of microarray based gene expression data. Our computational pipeline (Supplementary figure 4) infers a set of gene-specific TF activities (TFAs) and TF-specific TF concentrations (TFCs); identifies statistically significant up and down-regulation by utilizing TFAs and TFCs; outputs significant results in a format that makes it easy to visualize trends and patterns.

TFAs are the intensity of the interactions between a certain TF and its targets at a certain experimental point. TFCs must also be considered when evaluating TFAs, since a higher concentration of TF may induce more gene expression than a lower concentration (7). A joint analysis of TFAs and TFCs should provide more robust predictions that are closer to the complex reality of biology.

**Inference Model.** Genes and TFs must be precisely investigated for estimating TFA and TFC so we used a probabilistic variational inference model to estimate the concentration of each TF protein and the regulatory intensities of each TF and gene pair (8) . This requires gene expression measurements and connectivity data linking transcription factors to genes. The method obtains the TFAs and TFCs by regressing the gene expression data on the connection topology information. A Markov chain constructs the temporal structure of the data, and an efficient variational Expectation Maximization (EM) algorithm estimates the model parameters and posterior statistics. The model discussed in the paper by Sanguinetti et al. (8) has been applied to this study to investigate the changing of TF activities and concentration level.

**Estimation of Significant Differences (SigDiffs).** A set of TFAs for each gene and motif pair, and the associated TFCs, was obtained from the probabilistic variational inference model for each experimental condition. However, we were specifically interested in any TFAs which exhibit a SigDiff between the two conditions. This estimation process generated a list of TFAs between the two different experiments with SigDiffs. The SigDiff estimation from TFAs and TFCs required an equation. We assumed, based on the correlation of TFAs and TFCs (7), that a strong TFA of highly concentrated TF may be not the statistically significant. In contrast, a weak TFA of lowly concentrated TF may be a statistical significance. Therefore, the equation:


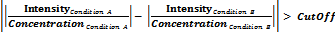


was used to find all SigDiffs of TFAs. We set the standard deviation as 2, thus all differences at the 95% confidence interval were considered significant.

**Results:**

**Microarray analysis:**

An overview of the analysis approach is shown in Fig S1.

Gene enrichment identified 469 (631 probe sets) that were differentially expressed in a statistically significant manner, which had q<0.1, and fold change > ±1.75 in any Gc treatment versus the control (some genes are represented on the array as multiple probesets, corresponding to specific sequences arranged along the expressed sequence). Fewer probesets passed this threshold for pulse versus control (240), than C100 or C200 versus control (411 and 497 respectively). No genes passed this stringent threshold in the direct comparisons between the different Gc deliveries. This confirms the PCA results above where the major difference in the dataset was found between control and the cortisol treatments.

Although no genes in the microarray analysis passed our initial, strict threshold for differential expression in pulse versus C100 there clearly appeared to be differences between the treatments, as suggested by the PCA results reported above. A volcano plot of the dataset highlighting cell adhesion genes is shown in Fig S2A, and cell cycle in Fig S2B. This analysis shows the statistical significance on the y axis against the fold difference between the groups on a log scale on the x axis. Therefore genes expressed more highly in response to pulse cortisol appear to the right of the y axis, with an indication of statistical significance given by height on the y axis. Relaxing the threshold to pvalue <0.05 and fc > 1.75 in the pulse versus C100 or pulse versus C200 results in 468 and 262 probesets respectively. DAVID analysis of these gene lists reported cell adhesion and membrane associated actin binding (cytoskeleton) as significantly over-represented (Benjamini corrected p-value < 0.01), indicating that these gene networks are differentially regulated by cortisol delivery kinetics.

**Table 1. Gene-specific primers used in real-time PCR**

Gene name Sequence (5'–3') Optimal Concn, nM Size, bp

MT1X Forward: GCACCTCCTGCAAGAAGAG 800 19

Reverse: GCACTTGTCTGACGTCCCT 800 19

ITGA10 Forward: GCAACCCTAGAACCCTGGT 400 19

Reverse: GATTTCCAGTAAGCATCAAGCA 400 22

MAOA Forward: GGTCTTGAAGTTCTGTTCTTATGC 800 24

Reverse: CCAATGACACAGCCTTTAAACT 800 22

ITGA5 Forward: AGACTCAGACATTGGCACCTAA 800 22

Reverse: TCCTAAATCAGGGTGAACTGG 800 21

FKBP5 Forward: CATGAACGAGTTTGAGTCAGC 400 21

Reverse: TCTGGCACATGGAGATCTG 400 19

FOXO1 Forward: CAATGGAACATCCCAAGAAGA 400 21

Reverse: CCAGCAGTTGAACAAGTCCA 400 20

MAP3K7 Forward: GAATGATTACTACCTCAGGACCAA 400 24

Reverse: TGGTATCTGTGGAATCATCAGG 400 22

PRUNE2 Forward: CATTAGTACTGTGAGCATGAACCTT 800 25

Reverse: AGTTACATTCCAGTTGCTCAGACT 800 24

NR3C1 Forward: ACTGTAAGCTATGGATGTTGCAC 800 23

Reverse: ATGAATGTGCGCTTTGGA 800 18

NR6A1 Forward: GAATGTTTCTCCCTCGTACCA 400 21

Reverse: AATAACGTCTGCTCGGCAT 400 19

IGFBP5 Forward: TTCAGATTCCGAGTTGCCT 400 19

Reverse: TGTACTGTGGTTATTGCTGTCTTC 400 24

COL7A1 Forward: CCCTGGCAGATGACTCACT 800 19

Reverse: CCATGTCATCACAGGCTTG 800 19

CD97 Forward: GCACCTGTGGCCAGTACTC 400 19

Reverse: CCAGCGCCTAGTCTCTTCA 400 19

GPR56 Forward: AACAAACACACACGGGTAGATT 800 22

Reverse: ATACCAGACTGAATGTTGGCA 400 21

ADAM12 Forward: CCCACTGTATCTAGGCAACATAGT 400 24

Reverse: TGTATTAGAGCTGGGTTCCCTT 400 22

GAPDH Forward: TCAACGACCACTTTGTCAAGCTCA 800 24

Reverse: GCTGGTGGTCCAGGGGTCTTACT 800 23

β-actin Forward: TTGTTACAGGAAGTCCCTTGCC 800 22

Reverse: ATGCTATCACCTCCCCTGTGTG 800 22

**References:**

1. Sommer P, Le Rouzic P, Gillingham H et al. Glucocorticoid receptor overexpression exerts an antisurvival effect on human small cell lung cancer cells. Oncogene 2007; 26(50):7111-7121.

2. Donn R, Berry A, Stevens A et al. Use of gene expression profiling to identify a novel glucocorticoid sensitivity determining gene, BMPRII. FASEB J 2007; 21(2):402-414.

3. Li C, Wong WH. Model-based analysis of oligonucleotide arrays: expression index computation and outlier detection. Proc Natl Acad Sci U S A 2001; 98(1):31-36.

4. Irizarry RA, Hobbs B, Collin F et al. Exploration, normalization, and summaries of high density oligonucleotide array probe level data. Biostatistics 2003; 4(2):249-264.

5. Smyth GK. Linear models and empirical bayes methods for assessing differential expression in microarray experiments. Stat Appl Genet Mol Biol 2004; 3:Article3.

6. Huang dW, Sherman BT, Lempicki RA. Systematic and integrative analysis of large gene lists using DAVID bioinformatics resources. Nat Protoc 2009; 4(1):44-57.

7. Driever W, Thoma G, Nusslein-Volhard C. Determination of spatial domains of zygotic gene expression in the Drosophila embryo by the affinity of binding sites for the bicoid morphogen. Nature 1989; 340(6232):363-367.

8. Sanguinetti G, Lawrence ND, Rattray M. Probabilistic inference of transcription factor concentrations and gene-specific regulatory activities. Bioinformatics 2006; 22(22):2775-2781.
