# Supplementary figures and images for: Ultradian Cortisol Pulsatility Encodes a Distinct, Biologically Important Signal
Source: PLoS One. 2011 Jan 18;6(1):e15766. doi: 10.1371/journal.pone.0015766 (PMC3022879; doi:10.1371/journal.pone.0015766)

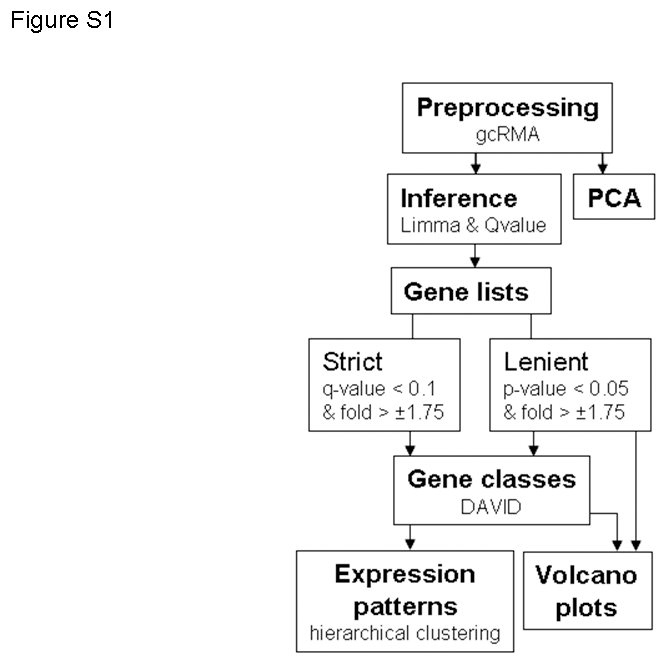

Supplement: Figure S1 — Gene expression array profiling: Flow chart showing data analysis strategy. Initial review of the data used gcRMA, then initial analysis was by Limma and q value, and principal component analysis. Two thresholds were applied for more detailed analysis, a strict, and a more lenient one. Resulting gene lists were analysed by DAVID for functional networks, and then by hierarchical clustering, and volcano plotting. (TIF) [file pone.0015766.s002.tif]

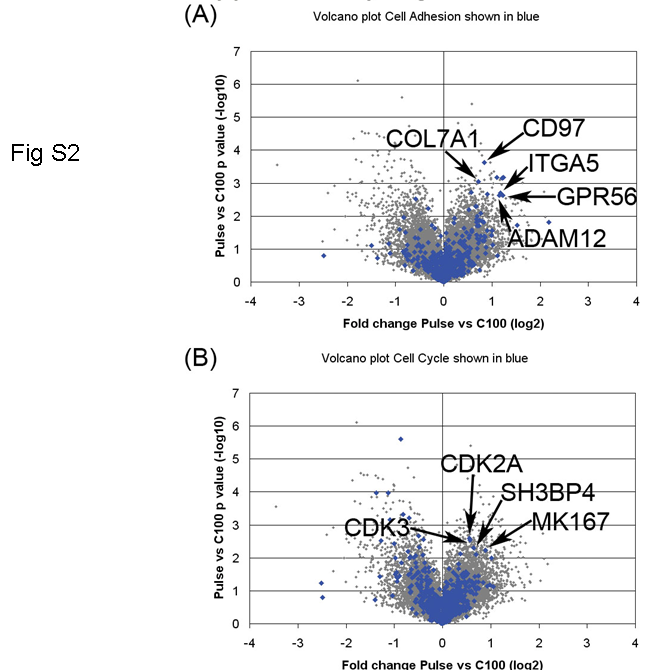

Supplement: Figure S2 — Volcano plots showing the magnitude of change in gene expression between pulse and continuous (C100) treatments. Genes expressed more highly under pulse conditions are shown to the right of the “y” axis, which depicts the (-Log10) of the P value. Cell adhesion genes (a – in blue) and cell cycle genes (b – in blue) that are differentially expressed between the two treatments are indicated. (TIF) [file pone.0015766.s003.tif]

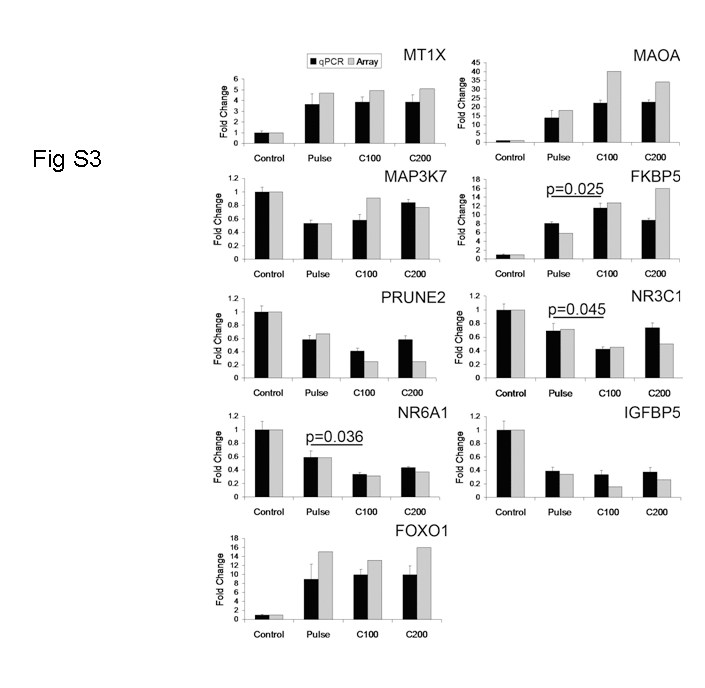

Supplement: Figure S3 — Quantitative RT-PCR validation of microarray data. Confirmation of the microarray results were sought using expression analysis of the indicated genes by quantitative real time PCR. All expression values were normalised to the average of β-actin and GAPDH. Data represents the mean and standard error of biological triplicate experiments. Exact P values are shown, analysis by Student's t test. (TIF) [file pone.0015766.s004.tif]

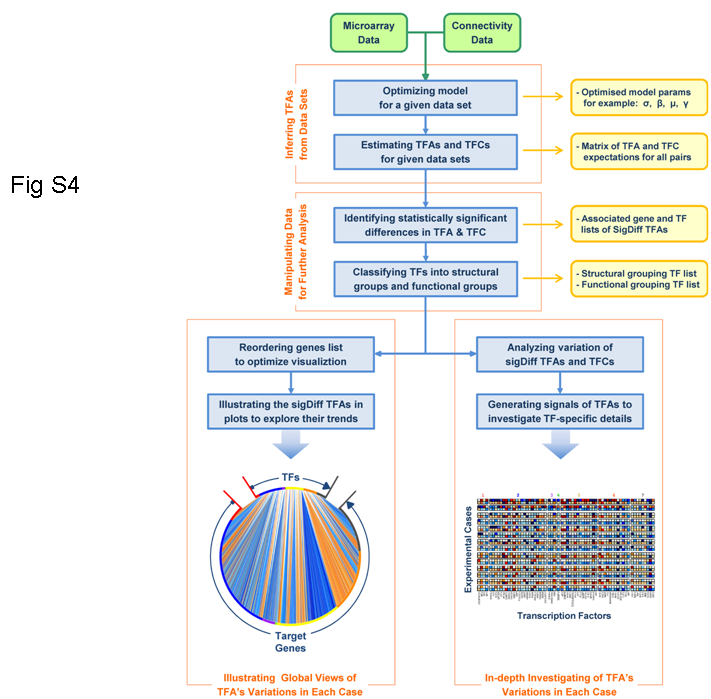

Supplement: Figure S4 — Overview of our systematic approach. The data analysis pipeline is composed of five parts: (1) RMA normalization of microarray data is performed, and a binary matrix containing connection topology is constructed. (2) The microarray data and connectivity data are utilized to infer TFAs and TFCs. (3) Once TFAs are estimated, the statistically SigDiffs are calculated. (4) SigDiff TFAs and TFs are analyzed and classified into structural groups and functional groups. (5) The SigDiff TFAs and TFCs are illustrated with TF-perspective views that show TFAs with associated TFCs by TF functional group and experimental condition and round limpet-like plots which show the TFA between individual TF and genes. (TIF) [file pone.0015766.s005.tif]
